# O.6.3-7 Participatory video as an evaluation method: lessons learned from a community-based participatory research project with women in difficult life situations

**DOI:** 10.1093/eurpub/ckad133.287

**Published:** 2023-09-11

**Authors:** Raluca Sommer, Alexandra Sauter, Stephanie Linder, Heiko Ziemainz, Karim Abu-Omar

**Affiliations:** Friedrich Alexander Universität Nürnberg-Erlangen; University of Regensburg, Germany; raluca.sommer@fau.de; Friedrich Alexander Universität Nürnberg-Erlangen; Friedrich Alexander Universität Nürnberg-Erlangen; Friedrich Alexander Universität Nürnberg-Erlangen

## Abstract

**Purpose:**

Visual research receives less attention compared to text-based research, especially in evaluating projects for physical activity (PA). This is surprising, since empowering individuals and communities to engage in the assessment tasks, and take decisions about the evaluation process has shown to have a positive impact on outcomes (Fetterman et al., 2018).

NU-BIG[1] set out to involve women in difficult life situations[2] (n = 6) in creating a video to reflect upon the long-term effects of a PA program. It implies “a collaborative approach in which people create a video about themselves to open spaces for learning, enable change and transformation” (Milne et al., 2012, pp.36).

**Methods:**

We conducted a qualitative study using a semi-standardized monitoring protocol, semi-structured interviews (n = 12), field notes (n = 4), and a reflexive focus group interview (n = 1) to investigate the process of making the video.

**Results:**

The video-making process lasted from November 2021 to July 2023. Along the way, researchers and the women involved faced numerous challenges.

For the women, deciding on doing a video resulted in insecurities and often seemed to rather inhibit their active participation. They needed sustained support and guidance from scientists (i.e., organizing meetings, providing content), as well as the help of a media professional to handle practical aspects. However, in later stages of the process, they also experienced dynamic learning, which improved their general media literacy, self-confidence and critical awareness.The women clearly voiced that the video was to be done on their terms. This put the involved researchers in a challenging situation. Researchers were forced to continuously reflect on their own role and identity in the process, and had to relinquish control. This required them to be flexible in e.g. the facilitation of meetings and a permanent ability to adapt to spontaneous situations and demands.

**Conclusions:**

The video enabled women to share how PA had influenced their lives, allowing them to reclaim their voices and combat feelings of marginalization. As an evaluation method, it can enable relevant stakeholders to gain valuable data, which can contribute to social change. Visual research methods should be given more importance, particularly in community-based projects with vulnerable population groups.

